# Exploring the Potential of Chatbots in Critical Care Nephrology

**DOI:** 10.3390/medicines10100058

**Published:** 2023-10-20

**Authors:** Supawadee Suppadungsuk, Charat Thongprayoon, Jing Miao, Pajaree Krisanapan, Fawad Qureshi, Kianoush Kashani, Wisit Cheungpasitporn

**Affiliations:** 1 Division of Nephrology and Hypertension, Department of Medicine, Mayo Clinic, Rochester, MN 55905, USA; s.suppadungsuk@hotmail.com (S.S.); miao.jing@mayo.edu (J.M.); pajaree_fai@hotmail.com (P.K.); qureshi.fawad@mayo.edu (F.Q.); kashani.kianoush@mayo.edu (K.K.); wcheungpasitporn@gmail.com (W.C.); 2 Chakri Naruebodindra Medical Institute, Faculty of Medicine Ramathibodi Hospital, Mahidol University, Samut Prakan 10540, Thailand; 3 Division of Nephrology and Hypertension, Thammasat University Hospital, Pathum Thani 12120, Thailand

**Keywords:** chatbots, artificial intelligence, critical care nephrology, nephrology

## Abstract

The exponential growth of artificial intelligence (AI) has allowed for its integration into multiple sectors, including, notably, healthcare. Chatbots have emerged as a pivotal resource for improving patient outcomes and assisting healthcare practitioners through various AI-based technologies. In critical care, kidney-related conditions play a significant role in determining patient outcomes. This article examines the potential for integrating chatbots into the workflows of critical care nephrology to optimize patient care. We detail their specific applications in critical care nephrology, such as managing acute kidney injury, alert systems, and continuous renal replacement therapy (CRRT); facilitating discussions around palliative care; and bolstering collaboration within a multidisciplinary team. Chatbots have the potential to augment real-time data availability, evaluate renal health, identify potential risk factors, build predictive models, and monitor patient progress. Moreover, they provide a platform for enhancing communication and education for both patients and healthcare providers, paving the way for enriched knowledge and honed professional skills. However, it is vital to recognize the inherent challenges and limitations when using chatbots in this domain. Here, we provide an in-depth exploration of the concerns tied to chatbots’ accuracy, dependability, data protection and security, transparency, potential algorithmic biases, and ethical implications in critical care nephrology. While human discernment and intervention are indispensable, especially in complex medical scenarios or intricate situations, the sustained advancements in AI signal that the integration of precision-engineered chatbot algorithms within critical care nephrology has considerable potential to elevate patient care and pivotal outcome metrics in the future.

## 1. Introduction

### 1.1. Definition and Overview of Chatbots

Chatbots have become a prominent implementation of AI in diverse domains, including customer service, healthcare, and e-commerce. These conversational agents, powered by AI, are designed to engage with users in a similar manner to human conversations [1,2].

The concept of chatbots originated in the mid-20th century with early developments in natural language processing and machine learning [3]. Notably, a precursor to modern chatbots, known as ELIZA, emerged in the 1960s as a program designed to simulate conversation with a Rogerian psychotherapist [4,5,6]. Since then, chatbot technology has undergone significant advancements driven by improvements in AI, machine learning techniques, and big data analytics [7,8]. The critical components of chatbots are as follows:

(1) Natural language processing (NLP) forms the foundation of chatbot technology, a subfield of AI that empowers machines to comprehend and process human language [9,10,11]. NLP encompasses several essential elements, including the following: (1) Text preprocessing involves preparing text inputs from a chatbot for subsequent analysis. This stage entails removing unnecessary punctuation, converting the text to lowercase, and segmenting sentences into individual words (tokenization) [12]. (2) Language understanding focuses on extracting meaning from the preprocessed text. Methods like named entity recognition and part-of-speech tagging, and sentiment analysis are employed to identify entities, categorize words, and ascertain the sentiment underlying the user’s input [13]. (3) Intent recognition aims to discern the user’s intention or the purpose behind their input. Machine learning algorithms are trained to identify intent by analyzing user text patterns, enabling the chatbot to determine the appropriate response or action [2].
(2) Machine learning plays a pivotal role in the development of chatbots, enabling them to learn from data and improve their performance [2,3,14]. In the context of chatbot development, several machine learning algorithms play pivotal roles: (1) Supervised learning: This approach involves training the chatbot using labeled datasets, where each input is paired with a predefined output. By learning from these examples, the algorithm can classify new inputs and generate appropriate responses. Supervised learning is particularly advantageous for tasks such as intent recognition and entity extraction [3]. (2) Unsupervised learning: Chatbots trained using this methodology rely on unlabeled datasets. The model is primarily built on self-learning derived from historical chat log data. This results in conversation interactions that are more diverse and natural compared to those generated through supervised learning. However, a potential drawback of unsupervised learning is the possibility of generating inaccurate results due to the absence of predefined outputs [15]. (3) Semi-supervised learning: This is a hybrid approach that combines elements of both supervised and unsupervised learning. It utilizes unlabeled data to cluster related information and subsequently labels these data to enhance the learning process [16]. (4) Reinforcement learning: This iterative methodology involves training the chatbot through a trial-and-error process [17]. The chatbot interacts with users, receives feedback on the quality of its responses, and adjusts its behavior based on this feedback. By maximizing the rewards received for desirable actions, reinforcement learning enables the chatbot to continuously refine and enhance its performance over time [3].
(3) Dialogue management focuses on governing the flow of conversation between the chatbot and the user [13,18]. It encompasses three key components, including the following: (1) State tracking involves maintaining context throughout the conversation, including the user’s current goal, preferences, and previous interactions. By preserving this information, the chatbot can provide more personalized and contextually relevant responses [19,20]. (2) Policy learning determines how the chatbot selects responses based on the current state of the conversation. Policy learning algorithms are trained to optimize the chatbot’s decision-making process, considering user satisfaction, system constraints, and task completion [13]. (3) Response generation is the process by which the chatbot generates an appropriate action or response. This can range from simple template-based responses to more sophisticated approaches, such as natural language generation (NLG) or neural-network-based models that generate responses from scratch [9].

Integration and deployment involve the various platforms and channels through which chatbots can be deployed, such as messaging apps, websites, or voice-based interfaces. Integration with existing systems, such as customer relationship management (CRM) tools or backend databases, enables chatbots to access relevant information and provide seamless user experiences [8,21]. Furthermore, continuous monitoring and improvement are critical for optimizing chatbot performance [22]. This involves gathering user feedback, analyzing conversation logs, and regularly updating the chatbot’s knowledge base and algorithms to refine its functionality and enhance user satisfaction [23,24].

### 1.2. Introduction to the Potential Utilization of Chatbots in Critical Care Nephrology

AI has made significant advancements in recent decades, particularly in its application within the healthcare sector [25,26,27,28,29]. In critical care settings, several multidisciplinary care teams play a crucial role in patient care. Nephrology has emerged as one of the most consulted in recent years. Acute kidney injury (AKI) incidence in critically ill patients has been reported to range from 30% to 57% [30,31,32]. AKI, renal complications, and dialysis intervention correlate with significantly higher morbidity and mortality. Therefore, it is essential to highlight the significance of critical care nephrology to improve patient outcomes. The integration of chatbots into this process could potentially enhance patient metrics.

Notable examples include OpenAI’s ChatGPT (powered by GPT-3.5 and 4.0), Microsoft’s Bing Chat (which utilizes GPT-4.0), and Google’s Bard AI (built on PaLM (Pathways Language Model)). These chatbots have the potential to revolutionize patient care, enhance clinical decision making, and improve healthcare outcomes [33,34,35,36].

In recent years, emerging technological innovations have brought transformative changes to various domains of medicine, and intensive care medicine (ICM) has been a prominent beneficiary of these advancements [37]. The relevance of innovative methods, particularly AI, in ICM has been underscored by the emergence of the COVID-19 pandemic, which has heightened the demand for intensive care unit (ICU) resources. Lu et al. [38] recently explored the potential advantages of incorporating expansive language models like ChatGPT and GPT-4 into the realm of intensive care medicine. Utilizing the advanced features of AI-driven chatbots, including models like ChatGPT and GPT-4, medical professionals may elevate patient care with comprehensive knowledge, precise data interpretation, and judicious decision making. By combining human proficiency with AI assistance, there is an opportunity to transform clinical approaches, optimize resource distribution, and significantly improve patient results in the realm of intensive care medicine.

In another article by Alhasan et al. [39], the challenges posed by pediatric respiratory viruses like RSV and metapneumovirus following the COVID-19 pandemic are examined. The authors highlight the role of ChatGPT in providing guidance for specialists in pediatric intensive care. ChatGPT’s advisories encompass adherence to established protocols, infection prevention strategies, vigilant patient monitoring, respiratory support, management of pre-existing conditions, accurate medication administration, holistic patient care, patient education, and self-care emphasis. While the study acknowledges the swiftness with which ChatGPT processes information and offers suggestions, it also emphasizes the importance of corroborating AI-generated guidelines with clinical discernment tailored to each patient. The potential of ChatGPT to offer guidance during evolving viral outbreaks is evident, though considerations regarding biases and inaccuracies warrant further exploration, highlighting the necessity for human specialists’ validation.

We, as critical care nephrologists, play a pivotal role in managing patients who are critically ill, particularly within the ICU setting. Among the challenges we encounter, a significant portion involves patients suffering from AKI, a condition associated with elevated mortality rates. Given this context, it is crucial to explore the utilization of chatbots for critically ill patients in the realm of critical care nephrology, with the aim of enhancing patient outcomes. While the integration of chatbots into critical care nephrology is not yet widespread, the concept of incorporating these AI-driven tools into our specialized field holds significant importance. Our expertise lies in caring for acutely ill patients grappling with acute kidney injury, many of whom require immediate renal replacement therapy. In this context, the application of AI-driven chatbots could offer substantial advantages. These chatbots could serve as complementary tools, aiding us in navigating complex clinical scenarios, analyzing patient data, and providing real-time insights that contribute to informed decision making.

As we navigate the evolving landscape of intensive care medicine, we recognize the potential applications of AI and their implications. Given our unique position as critical care nephrologists, it is vital to carefully consider and explore how chatbots can augment our ability to provide optimal care to critically ill patients with AKI. The integration of these AI tools has the potential to bridge gaps in knowledge, streamline processes, and ultimately lead to improved patient outcomes. In our pursuit of innovation, complemented by our deep-rooted expertise, we aim to combine human discernment with AI assistance to elevate the standard of care in the ICU. Introducing chatbots to critical care nephrology can present a multitude of benefits for patients, healthcare professionals, and the overall healthcare infrastructure [40,41,42]. One of the primary advantages is the enhancement of healthcare accessibility. Chatbots, which operate 24/7, can promptly address a wide range of patient inquiries and concerns, ensuring they receive relevant information even beyond conventional clinical hours. Additionally, these chatbots can potentially serve as valuable tools for healthcare providers, aiding them in making judicious decisions [43,44,45,46,47]. Through the analysis of extensive patient data, including laboratory results, vital signs, and medical histories, chatbots can identify patterns, notify healthcare providers of potential complications, and suggest appropriate interventions [48,49]. This real-time analysis has the potential to save crucial time, particularly in critical situations where prompt decision making is vital [50]. Additionally, chatbots could serve as valuable educational tools for patients and healthcare professionals [51]. By engaging in personalized interactions, chatbots may provide tailored education regarding patients’ conditions, treatment options, and self-management strategies [52]. For healthcare professionals, chatbots may offer access to current medical literature, guidelines, and protocols, enabling them to stay informed and deliver evidence-based care [53].

## 2. The Future Imperative: Why We Need Chatbots in Critical Care Nephrology

**Figure 1:**
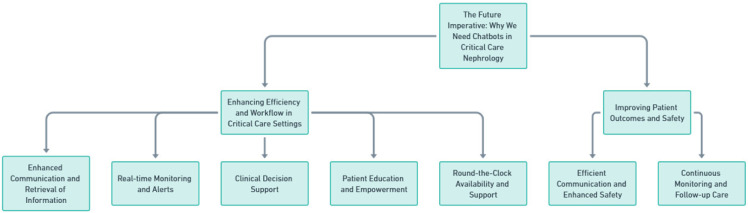
Structured overview of the significance of chatbots in the future of critical care nephrology.

As medical practices progress, adopting technological advancements transitions from being an added advantage to a fundamental requirement. Within the domain of critical care nephrology, such progress is evident. The escalating intricacy of patient management, combined with the persistent requirement for prompt and accurate actions, amplifies the imperative for novel strategies. Chatbots, which are conversational AI-enabled interfaces, are on the cusp of transforming the modus operandi of patient care in this niche sector. Their promise in augmenting operational efficiency, refining communication protocols, and elevating patient wellbeing categorizes them as essential apparatuses for the imminent future of critical care nephrology (Figure 1).

### 2.1. Enhancing Efficiency and Workflow in Critical Care Settings

Chatbots have emerged as potent instruments in critical care nephrology, where prompt decision making and effective communication are imperative. These AI-driven conversational agents have been designed to interact with healthcare professionals, patients, and their families, providing real-time aid and support. By integrating well-designed chatbots, critical care nephrology can realize enhanced efficiency, streamlined workflows, and improved patient care outcomes.

- Enhanced Communication and Retrieval of Information:

Healthcare professionals in critical care settings often encounter challenge accessing pertinent information promptly [54]. Chatbots can tackle this issue by acting as virtual assistants, granting immediate access to medical databases, guidelines, and patient records [42]. They can potentially retrieve laboratory results, medication details, and historical data, thereby equipping healthcare providers with comprehensive and up-to-date information that is readily available. Chatbots facilitate quicker decision making by streamlining communication and retrieving information, ultimately improving patient outcomes [55].

- Real-time Monitoring and Alerts:

The continuous monitoring of patients’ vital signs and laboratory parameters is crucial in critical care nephrology. Chatbots can be seamlessly integrated with electronic health records (EHRs) and monitoring devices, allowing real-time data aggregation [56,57,58]. Through the analysis of such data, chatbots can promptly identify anomalies or trends that may necessitate immediate attention [59]. They can promptly notify healthcare providers of critical values, changes in patient condition, or potential complications, thereby ensuring timely interventions and minimizing the risk of adverse events [60].

- Clinical Decision Support:

In care nephrology, making clinical decisions can be a common challenge. Chatbots, which are equipped with algorithms, offer evidence-based recommendations and clinical decision support [61,62]. They assist healthcare professionals in interpreting laboratory results, selecting tests, and determining the most effective treatment strategies [59]. By incorporating guidelines and best practices, chatbots may serve as resources that reduce errors and variations in patient care [63]. This enhancement optimizes the efficiency of healthcare providers and promotes standardized, high-quality patient management.

- Patient Education and Empowerment:

The education and active engagement of patients and their families are pivotal in critical care nephrology. Chatbots can potentially serve as virtual educators, delivering personalized educational materials, medication instructions, and lifestyle recommendations [64,65]. They can answer frequently asked questions, offer reminders for medication adherence and follow-up appointments, and support patients in managing chronic kidney conditions [65,66,67]. By empowering patients with knowledge and resources, chatbots improve patient satisfaction, compliance, and overall outcomes [68].

- Round-the-Clock Availability and Support:

Critical care settings necessitate continuous monitoring and immediate responses. Chatbots offer 24/7 availability, ensuring healthcare professionals can access support and information at any time [69]. They may assist in patient triaging, provide preliminary assessments, and offer initial guidance during emergencies [70]. This constant availability reduces the burden on healthcare providers, particularly during non-office hours, and facilitates efficient resource utilization [42,71].

### 2.2. Improving Patient Outcomes and Safety

- Efficient Communication and Enhanced Safety:

Effective and timely communication is crucial in critical care settings to ensure patient safety. Chatbots streamline communication among healthcare providers, enabling swift information exchange and facilitating collaboration [70]. They assist in organizing and prioritizing tasks, enhancing the efficiency of medical teams in managing their workload [42]. Additionally, chatbots can issue real-time alerts and notifications concerning critical laboratory results or medication interactions, ensuring prompt attention and minimizing the risk of adverse events [72]. By improving communication and safety, chatbots contribute to providing higher-quality care in critical care nephrology.

- Continuous Monitoring and Follow-up Care:

Following discharge, patients with kidney diseases require ongoing monitoring and follow-up care [70]. In this domain of critical care nephrology, chatbots assume a pivotal role. They can conduct remote monitoring, capture pertinent patient data, and identify deviations from the baseline [73]. Moreover, chatbots can facilitate virtual consultations, allowing patients to communicate with healthcare professionals, explain symptoms or concerns, and receive appropriate advice without needing in-person appointments [70,73]. This seamless continuity of care enables early detection of complications and timely interventions, resulting in improved patient outcomes and reduced readmission rates.

## 3. Features of Chatbots in Critical Care Nephrology

**Figure 2:**
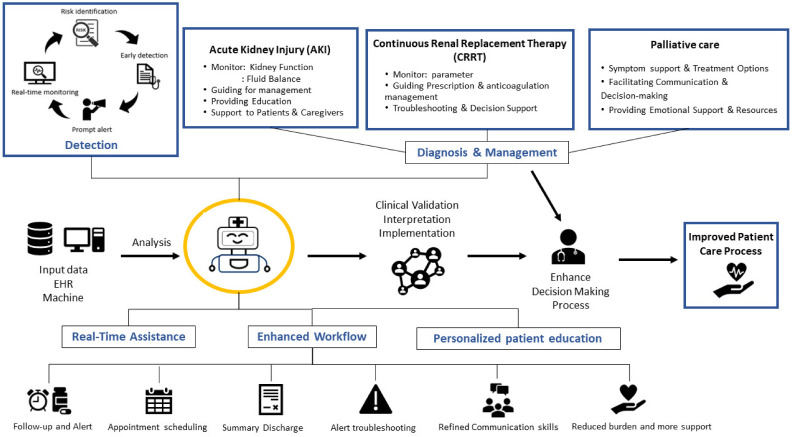
Features and potential uses for chatbots in critical care nephrology.

Chatbot technology, which is driven by AI algorithms, has gained considerable attention due to its potential applications across various sectors, including healthcare [74,75]. In critical care nephrology, chatbots possess unique features that can revolutionize patient care, enhance clinical decision making, and improve overall outcomes. This section explores the specific characteristics of chatbots in critical care nephrology and their potential benefits (Figure 2).

### 3.1. Real-Time Assistance and Accessibility

Chatbots can potentially play a pivotal role in critical care nephrology by offering immediate assistance to both patients and healthcare providers [76]. With the backing of advanced AI algorithms, these chatbots provide continuous support, promptly responding to frequent patient inquiries and worries. This capability considerably amplifies healthcare accessibility, ensuring that patients obtain relevant information and backing even beyond traditional clinical operating times [52]. For those grappling with kidney-related challenges, chatbots may emerge as indispensable assets, delivering instant counsel and easing apprehension.

### 3.2. Data Analysis and Decision Support

The capability of chatbots to analyze extensive patient data [77], including laboratory results, vital signs, and medical histories, is a notable feature. Through AI algorithms, chatbots can identify patterns, detect abnormalities, and provide valuable insights to healthcare providers [49,78]. This feature is especially valuable in critical care nephrology, enabling chatbots to alert healthcare providers to potential complications and suggest appropriate interventions [41]. Real-time data analysis and decision support by chatbots can save crucial time, leading to more-informed decision making, particularly in critical situations where swift actions are vital.

### 3.3. Personalized Patient Education

Chatbots can be used as tools for providing personalized patient education on various health topics, including conditions, treatment options, and self-management strategies [79,80]. By engaging in conversations with patients, chatbots gather relevant information and offer tailored educational content. A systematic review of randomized control trials on the effectiveness of chatbots in healthcare intervention demonstrated that implementing chatbots is feasible and positively affects physical functioning, healthy lifestyle, mental health, and psychosocial outcomes. Furthermore, chatbots could support patient health before or after medical treatment [35]. In critical care nephrology, this feature proves beneficial for patients with kidney-related issues who require comprehensive understanding and guidance [81]. Such tools can provide detailed information on medication compliance, dietary limitations, and necessary lifestyle changes. This enables patients to actively engage in their healthcare, which can lead to improved overall health results [82].

### 3.4. Access to Medical Literature and Guidelines

Healthcare professionals in critical care nephrology can leverage chatbots to access up-to-date medical literature, guidelines, and protocols [83]. By integrating chatbots with relevant databases and resources, healthcare providers can quickly retrieve accurate and evidence-based information [84,85]. This feature ensures that healthcare professionals can access the latest research, treatment guidelines, and best practices, enabling them to make informed decisions and deliver evidence-based care. It also supports continuous professional development by facilitating easy access to educational materials.

### 3.5. Language Processing and Multilingual Support

Chatbots equipped with advanced natural language processing capabilities excel in understanding and responding to patients’ queries and concerns effectively [74,86]. In critical care nephrology, where clear communication is crucial, this feature becomes highly valuable. Chatbots can interpret complex medical terminologies and provide explanations in layman’s terms, enhancing patient understanding and engagement. Furthermore, chatbots can offer multilingual support, overcoming language barriers and ensuring that patients from diverse backgrounds can access the care they need [87].

### 3.6. Integration with EHRs

To maximize their effectiveness, chatbots can seamlessly integrate with EHR systems. This integration allows chatbots to access patients’ medical histories, test results, and treatment plans, compiling a comprehensive patient health status [88]. By retrieving and analyzing relevant data in real time, chatbots can offer personalized recommendations and reminders, improving care coordination and treatment adherence. This feature streamlines the workflow for healthcare providers, enabling them to make more informed decisions based on up-to-date patient information [38,89].

### 3.7. Privacy and Security Measures

Given the sensitive nature of healthcare data, implementing robust privacy and security measures is crucial when integrating chatbots [90]. Measures such as robust encryption, authentication mechanisms, and strict data governance protocols should be in place to preserve patient privacy and maintain compliance with relevant regulations [71]. Ensuring data confidentiality and implementing secure data storage practices are essential to building trust among patients and healthcare providers.

## 4. Challenges and Limitations of Chatbot Implementation

### 4.1. Addressing Privacy and Security Concerns

**Figure 3:**
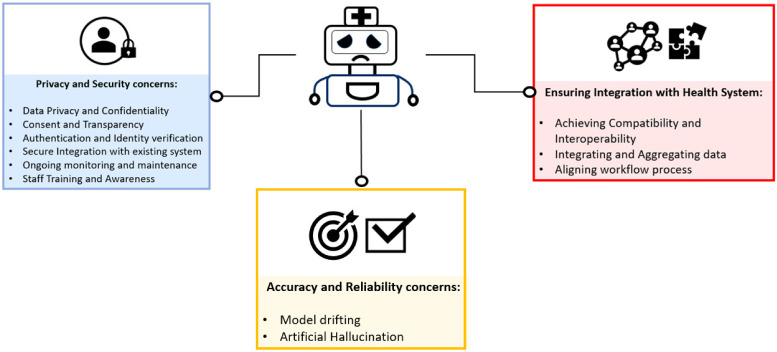
Challenges and limitations of chatbot implementation.

Although there are advantages to implementing chatbots in healthcare, there are also challenges and limitations that require attention [91]. A primary concern is ensuring the privacy and security of patient data [71]. The subsequent section outlines the challenges associated with privacy and security in chatbot implementation (Figure 3 and Table 1).

- Data Privacy and Confidentiality:

When chatbots are used in healthcare settings, they interact with patients, gather health information, and process sensitive data. Maintaining patient privacy and confidentiality is crucial in healthcare practices [91,92]. Healthcare organizations must ensure that their chatbot platforms comply with data protection regulations such as HIPAA in the United States or GDPR in the European Union [71,93]. This involves implementing encryption mechanisms, secure data storage practices, and strict access controls to prevent unauthorized breaches or access [90].

- Consent and Transparency:

Obtaining clear and informed consent plays a pivotal role in chatbot interactions. Patients should be aware of what data are being collected, why they are being collected, and their rights regarding their information [90,93]. Chatbots should prioritize transparency by providing explanations of how they collect and use data. This can be achieved through privacy policies and consent forms. It is crucial for healthcare organizations to allow patients the option to revoke their consent or opt out of sharing their data if they wish to do so. By communicating and considering patient preferences, trust can be fostered and ethical practices can be upheld when implementing chatbots [94].

- Authentication and Identity Verification:

Ensuring that users engaging with chatbots are authenticated is essential in preventing access or the disclosure of sensitive information. Robust authentication mechanisms, such as dual factor identification or biometric verification, should be implemented to ensure that only authorized individuals interact with the chatbot platform [93]. Healthcare organizations should also establish user identification protocols to minimize risks associated with impersonation or the unauthorized use of patient accounts [91].

- Secure Integration with Existing Systems:

Integrating chatbots into existing healthcare systems like EHRs or laboratory databases requires careful attention to security measures [94]. Comprehensive security assessments and vulnerability testing should be conducted by healthcare companies to identify and address any gaps or vulnerabilities in the integration process [71,90]. To ensure the security of data transmitted and stored between the chatbot and other systems, it is crucial to implement application programming interfaces (APIs) and utilize industry standard security protocols such as Transport Layer Security (TLS) or Secure Sockets Layer (SSL) [93].

- Ongoing Monitoring and Maintenance:

Regular monitoring and maintenance efforts are necessary to maintain a chatbot ecosystem. Healthcare organizations should keep an eye on chatbot activities to identify any abnormal behavior that might indicate security breaches or data compromise. By implementing logging mechanisms and intrusion detection systems, potential security incidents can be promptly identified and addressed [94]. Additionally, it is essential to keep the chatbot platform and its underlying infrastructure up to date with security patches and updates in order to mitigate emerging vulnerabilities and protect against evolving security threats [91].

- Staff Training and Awareness:

Training and raising awareness among staff members play a role in ensuring chatbot system security [83]. Healthcare organizations should prioritize providing training to their staff, including chatbot developers, administrators, and healthcare professionals, on practices for maintaining data privacy and security [93]. This includes educating them about coding practices and proper password management, as well as raising awareness about social engineering attacks like phishing attempts [95]. Having an informed workforce that remains vigilant serves as the primary defense against potential security breaches.

### 4.2. Accuracy and Reliability Concerns

- Model drift

Ensuring the accuracy and reliability of data when incorporating chatbots into patient care metrics can be challenging due to the phenomenon of model drifting. In the real world, data are constantly changing due to environmental factors and the growth of innovative input data over time. Model drift, or model decay, refers to the decline in machine learning performance over time. The data the model was trained on could have low accuracy, become outdated, or no longer be relevant to the current situation [96]. There are two types of model drift: (1) Concept drift happens when the input and target variable changes can be gradual, sudden, incremental, or recurring. (2) Data drift happens when input data change over time, such as the distribution of ages [96,97]. As a consequence of model drifting, there is a possibility that the algorithm powering the chatbot AI may generate inaccurate data. Implementing rigorous testing and validation protocols, as well as ongoing monitoring, retraining, and maintenance of data quality, can minimize the risks associated with these issues and continue to advance the field of chatbots in healthcare in a responsible and sustainable manner.

- Artificial hallucination

The phenomenon of AI or chatbots generating plausible information that is not aligned with reality has been described as artificial hallucination [98,99]. Hallucinations in natural language models can result from decoding errors, biases from previously generated data, or model encodes. It is crucial to address these sources to ensure accurate and relevant output. Previous studies in medical content and nephrology demonstrated that chatbots generate fabricated references 30–40% [99,100,101]. The references given by the chatbot were only 20% authentic. Additionally, the chatbot provided nephrology references with incorrect digital object identifiers (DOIs) and links 54% and 68% of the time, respectively. Notably, 24% of references in AKI fields were authentic, while references on hemodialysis were fabricated 60% of the time, and only 4% were genuine [100]. Mitigating hallucinations in chatbots by ensuring the system is well-trained and tested using a diverse and representative dataset is crucial. Furthermore, incorporating monitoring and using critical thinking before implementing data might alleviate this issue. 

### 4.3. Ensuring Integration with Existing Healthcare Systems

- Achieving Compatibility and Interoperability:

Integrating chatbots with existing healthcare systems provides a considerable issue in assuring compatibility and interoperability. Healthcare organizations commonly employ a variety of systems, including EHRs, laboratory information systems, and appointment scheduling platforms [8,25]. These systems often operate on distinct technologies, data formats, and standards, creating complexities in the integration process [3,35]. To address this challenge, conducting a comprehensive analysis of the existing systems and determining the required data exchange formats and protocols is imperative. Facilitating seamless communication between the chatbot and other systems can be accomplished by developing application programming interfaces (APIs). These APIs act as intermediaries that enable standardized and secure data exchange. Leveraging industry-standard interoperability frameworks like HL7 FHIR (Fast Healthcare Interoperability Resources) further enhances compatibility and streamlines the integration process.

- Integrating and Aggregating Data:

Another obstacle encountered when integrating chatbots with existing healthcare systems is integrating and aggregating data from multiple sources. Chatbots rely on accurate, up-to-date patient information to provide personalized and relevant responses [8,102]. However, patient data are often distributed across various systems, making real-time access and aggregation challenging. To overcome this challenge, organizations should implement data integration solutions capable of consolidating information from disparate sources [3,20]. Data warehouses or lakes can be central repositories for storing and organizing data from multiple systems, facilitating a unified and comprehensive view [52]. Data integration tools and techniques, such as data mapping and transformation, enable data extraction, processing, and loading into the chatbot system [103]. Additionally, implementing real-time data synchronization mechanisms ensures that the chatbot can access the most recent patient information.

- Aligning Workflows and Processes:

A critical consideration when integrating chatbots with existing healthcare systems is the alignment of workflows and processes. Chatbots need to integrate seamlessly into established clinical workflows without disrupting the routines of healthcare providers [25]. Failure to align with existing processes may result in resistance from healthcare professionals and hinder the adoption of chatbot technology [52]. To ensure successful integration, a thorough analysis of existing workflows is necessary. Identifying key touchpoints where chatbots can add value and automating repetitive tasks can enhance efficiency and acceptance among healthcare providers [8,86]. Involving healthcare providers in the design and development process helps address their concerns and ensures that the functionalities of the chatbot align with their specific needs.

## 5. Potential Applications of Chatbots in Critical Care Nephrology

**Figure 4:**
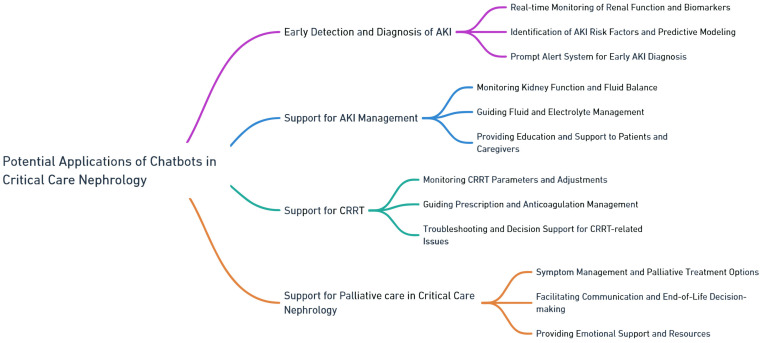
Potential applications of chatbots in critical care nephrology.

In this section, we explore the diverse and impactful potential applications of chatbots within the domain of critical care nephrology. As the management of kidney-related conditions in critically ill patients becomes increasingly complex, chatbots offer a potential novel approach to addressing various challenges and improving patient outcomes (Figure 4).

### 5.1. Early Detection and Diagnosis of AKI

Critical care nephrology is a specialized discipline involving the management of kidney-related conditions in critically ill patients. The implementation of chatbots in critical care nephrology has demonstrated potential in enhancing patient outcomes by enabling the early detection and diagnosis of AKI [48,72,104]. This section will explore the unique uses of chatbots in critical care nephrology, such as the real-time monitoring of renal function and biomarkers, the identification of AKI risk factors, and the development of a prompt alert system for early AKI diagnosis.

#### 5.1.1. Real-Time Monitoring of Renal Function and Biomarkers

The integration of chatbots offers promising avenues in the continuous monitoring of renal function and biomarkers for critically ill patients. With seamless connections to EHRs and laboratory systems, chatbots are well-positioned to collect and interpret real-time data, which includes serum creatinine levels [105,106], urine output [77], and other relevant parameters [50]. Through advanced NLP algorithms, chatbots hold the potential to translate these data into actionable insights, offering healthcare providers immediate updates and aiding in the early identification of AKI [107].

By actively monitoring renal function and biomarkers in real time [54,106], chatbots present healthcare providers with the opportunity to observe shifts and swiftly pinpoint irregularities. For example, should a patient’s creatinine levels surpass a specified limit, the chatbot might produce an alert, suggesting that healthcare professionals delve deeper and perhaps consider timely interventions to curb the progression of AKI [60].

#### 5.1.2. Identification of AKI Risk Factors and Predictive Modeling

Chatbots offer the potential to aid in pinpointing risk factors linked with AKI by accumulating patient-centric data and applying predictive modeling algorithms. Through dialogues with patients or caregivers, chatbots gather details about medical history, comorbidities, medications, and physiological parameters [78,106,108]. Utilizing machine learning techniques, chatbots can potentially analyze this information to discern risk factors leading to AKI development.

Employing predictive modeling, chatbots could produce risk scores or probabilities that denote the chance of AKI manifestation in critically ill patients [57]. This information equips healthcare providers with valuable insights to prioritize interventions, allocate resources effectively, and implement preventive measures to mitigate the risk of AKI [60,106,109]. For example, should a chatbot identify a patient with several AKI risk factors, it might suggest that healthcare professionals consider interventions like fine-tuning fluid management, recalibrating medication dosages, or adopting renal protective approaches.

#### 5.1.3. Prompt Alert System for Early AKI Diagnosis

The timely diagnosis of AKI poses a significant challenge in its management. Chatbots address this challenge by serving as prompt alert systems for the early detection of AKI. Through uninterrupted monitoring of patient data, encompassing renal function parameters and AKI risk determinants, chatbots might generate notifications upon specific criteria being fulfilled or certain thresholds being reached [43].

When a chatbot identifies an abnormal trend or a combination of risk factors indicative of potential AKI, it could immediately inform healthcare providers. This early alert system empowers healthcare teams to initiate diagnostic evaluations, implement appropriate interventions, and prevent further renal damage [47,56,58]. Moreover, chatbots might enhance decision making by recommending specific diagnostic procedures or strategies, all rooted in evidence-backed guidelines or institutional directives [60,110,111].

### 5.2. Support for AKI Management

AKI is a critical condition characterized by the sudden loss of kidney function, leading to the accumulation of waste products and imbalances in bodily fluids. Kidney replacement therapy, such as dialysis or CRRT, is crucial in managing AKI. The integration of chatbots in supporting AKI and CRRT management holds promise with regard to amplifying patient outcomes by assisting in various aspects of care [112,113]. This section explores the specific applications of chatbots within the context of AKI and CRRT management.

#### 5.2.1. Monitoring Kidney Function and Fluid Balance

Chatbots offer the possibility of aiding in the observation of kidney function and fluid equilibrium in AKI patients. Through seamless connections with electronic health records and laboratory systems, chatbots might gather and interpret real-time data points, encompassing serum creatinine levels, urine production, and fluid consumption and excretion [77]. With such data at their disposal, chatbots are well-poised to provide healthcare professionals with consistent updates on kidney functionality trends and fluid conditions [41].

Employing machine learning algorithms, chatbots could discern patterns and departures from typical benchmarks, alerting healthcare experts to crucial shifts or the exacerbation of AKI [43]. Such insights equip medical teams with the foundation for swift response measures, be it modulating fluid treatments or commencing kidney replacement therapies, aiming to avert complications and enhance patient results [114].

#### 5.2.2. Guiding Fluid and Electrolyte Management

Fluid and electrolyte management is critical to AKI care. Chatbots present the opportunity to bolster healthcare providers’ decision-making processes concerning fluid balance, electrolyte substitution, and medication modifications.

By analyzing patient-centric data regarding fluid consumption and expulsion, electrolyte metrics, and medication profiles, chatbots might propose strategies for fluid and electrolyte management [41,115]. These digital assistants could recommend suitable fluid revival volumes, the nature of fluids (for instance, isotonic or colloid), and the pace of dispensation, all tailored to the patient’s clinical state and distinct requirements.

In addition, chatbots hold the potential to provide guidance regarding electrolyte replenishment, such as recalibrating potassium or sodium concentrations, with due regard to renal performance and broader patient stability. Such insights play a role in averting electrolyte disparities and facilitating the reestablishment of regular physiological benchmarks [113].

#### 5.2.3. Providing Education and Support to Patients and Caregivers

Chatbots may act as invaluable educational platforms for AKI patients and their caregivers, delivering guidance and backing throughout the care journey. These digital assistants might offer tailored counsel on kidney health [115,116], lifestyle modifications, and self-care practices to promote recovery or prevent further kidney damage [66,67,117].

**Figure 5:**
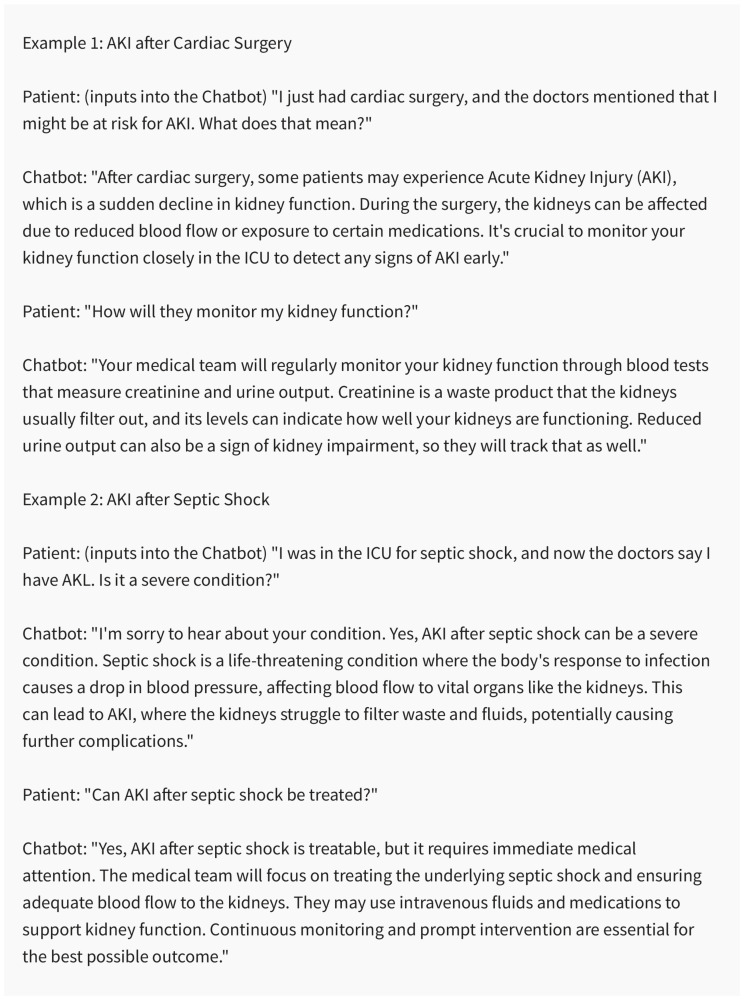
Chatbots serving as valuable educational tools for AKI patients. Created using ChatGPT 3.5.

With the integration of evidence-rooted resources and instructive content [68], chatbots may potentially educate patients and caregivers about AKI causes, symptoms, and the importance of adherence to prescribed treatments [70]. Furthermore, chatbots might oversee medication oversight, elucidating the correct deployment and probable adverse reactions of drugs pivotal to AKI treatment (Figure 5).

Furthermore, chatbots might present timely reminders for subsequent appointments, laboratory tests, and medication schedules, promoting patient engagement and compliance with the recommended treatment plan [41,115,117]. These digital assistants are well-placed to tackle patient questions, soothe anxieties, and extend emotional backing, augmenting the holistic patient journey and paving the way for improved results in AKI management [118].

### 5.3. Support for CRRT

CRRT is a widely utilized method for kidney replacement therapy in the management of AKI and other kidney-related conditions [119,120]. The inclusion of chatbots in the orchestration of CRRT management holds promise in amplifying patient outcomes, providing support throughout diverse care dimensions [54,119,120]. This section examines potential the specific development applications of chatbots within the context of CRRT management.

#### 5.3.1. Monitoring CRRT Parameters and Adjustments

In the context of CRRT, chatbots could play a crucial role in monitoring parameters and guiding adjustments to optimize treatment. By seamlessly integrating with CRRT machines and electronic health records, these bots have the potential to collect real-time data on critical factors like blood flow rates, dialysate flow rates, ultrafiltration rates, and other relevant parameters [121].

Leveraging machine learning algorithms, chatbots have the potential capacity to perpetually interpret data, positioning themselves to supply healthcare professionals with up-to-date information regarding the efficacy of CRRT. Such chatbots might discern patterns, detect deviations from desired targets, and promptly alert healthcare providers to significant changes or potential issues that may arise [54,114].

By actively monitoring CRRT parameters, chatbots might assist healthcare teams in undertaking timely interventions by providing data guidance support, such as fine-tuning blood flow rates, adjusting dialysate composition, or modifying ultrafiltration rates. This proactive approach helps maintain proper fluid and electrolyte balance, optimize solute clearance, and minimize the risk of complications associated with CRRT.

#### 5.3.2. Guiding Prescription and Anticoagulation Management

Chatbots might emerge as instrumental assets in aiding healthcare providers with orchestrating prescription and anticoagulation management for CRRT. By accessing patient-centric data, which includes weight, clinical status, and laboratory findings, chatbots hold the potential to suggest prescription configurations, such as suitable dosages and therapeutic durations [44,120].

Chen et al. [44] demonstrated the good performance and feasibility of using AI deep neural network models to detect and determine how to adjust the dose of regional citrate anticoagulant (RCA) to avoid citric acid overdose in CRRT patients by conducting an analysis of patient-specific parameters, such as coagulation profiles and bleeding risk factors. The overall accuracy of the neural network models was 90.77%. Even in anticoagulant-free patients, Zhang et al. validated and used AI to guide and predict filter lifespan among CRRT patients [122]. The findings showed that the internal validation model and area under the curve were 0.8 (95% CI (0.74–0.87) and 0.82 (95% confidence interval (CI) (0.76–0.88)), respectively.

Such chatbot assistance ensures the safe and effective delivery of CRRT, thereby minimizing the risk of clotting or bleeding complications arising from insufficient or excessive anticoagulation.

#### 5.3.3. Facilitating Troubleshooting and Providing Decision Support for CRRT-Related Issues

**Figure 6:**
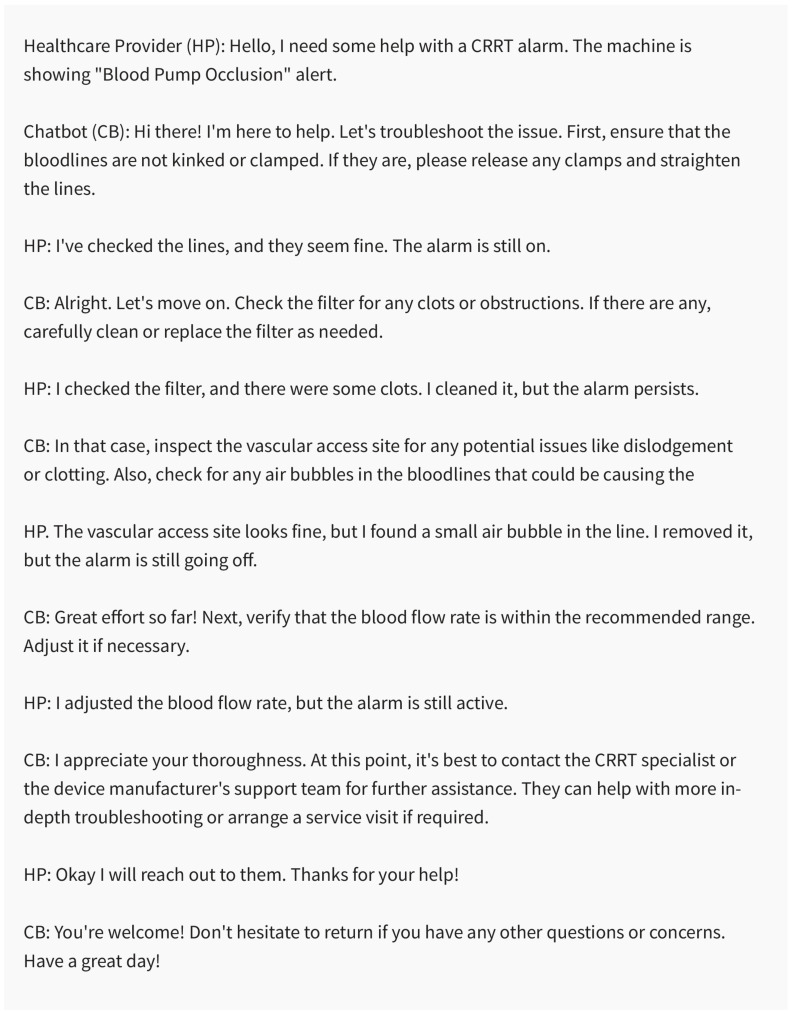
The chatbot provides troubleshooting support for the CRRT alarm, and the healthcare provider follows the recommended steps to resolve the issue. Created using ChatGPT 3.5.

Chatbots are potentially valuable sources of troubleshooting support and decision-making assistance for CRRT-related issues (Figure 6). In instances of alarms or technical difficulties with CRRT machines, chatbots might offer sequential guidance for foundational troubleshooting and issue mitigation.

Furthermore, chatbots can act as decision support tools by integrating clinical guidelines and expert knowledge. When confronted with complex situations or uncertainties in CRRT management, healthcare providers might turn to chatbots to acquire evidence-rooted suggestions and counsel.

For example, if an adjustment to the CRRT prescription is necessitated due to changing patient conditions or the need to address specific complications, chatbots might proffer insights on fitting alterations grounded in optimal practices and clinical standards. Table 2 depicts interactions between a healthcare provider and a chatbot for CRRT troubleshooting.

Furthermore, chatbots hold the potential to aid in determining the ideal span for CRRT. Through analysis of patient-specific factors such as fluid balance, kidney function recovery, and clinical stability, chatbots might guide healthcare professionals in discerning the right moment to cease CRRT and shift to other renal replacement therapies or supportive care strategies.

### 5.4. Support for Palliative Care in Critical Care Nephrology

The deployment of chatbots presents distinct merits and backup options within the realm of palliative care in critical care nephrology [45]. These automated dialogue facilitators have the capacity to elevate patient journeys and proffer invaluable assets [123]. Below is an exploration of the roles chatbots might play within the aforementioned spheres of palliative care.

#### 5.4.1. Assisting with Symptom Management and Palliative Treatment Options

**Figure 7:**
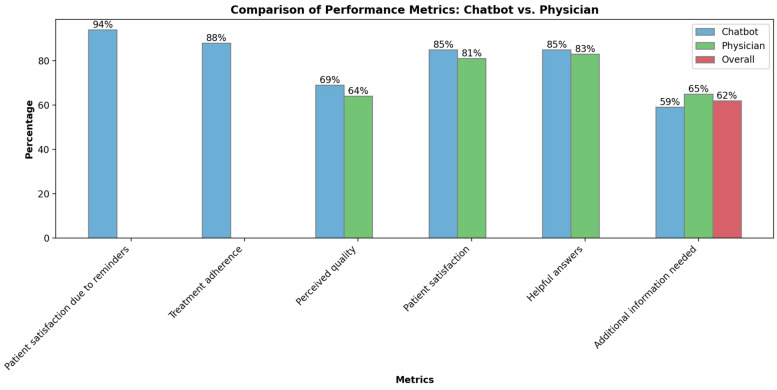
Performance metric comparisons between chatbots and physicians.

Chatbots are poised to dispense tailored data and backing for symptom oversight [73,124]. In the previously mentioned study, which investigated the interaction between 958 cancer patients and chatbots in monitoring patients’ symptoms and concerns, 132,970 message interactions were observed per month. The study showed that receiving medical reminders and accessing information via chatbots positively impacted patient satisfaction by 94%. Additionally, the chatbots helped and supported patients to effectively follow their treatment, with a success rate of 88% [79]. Another noninferiority RCT study compared chatbots and physicians regarding ability to give patients information and was conducted on 141 breast cancer patients. The findings revealed that the perceived quality of the answer by the chatbot was found to be non-inferior to the scores of the physicians. The success rates of the chatbot and physician groups were 69% and 64%, respectively (Figure 7). Patient satisfaction rates were 85% and 81% for the chatbot and physician groups, and the rates of helpful answers were 85% and 83%, respectively. The findings revealed that 62% of patients overall needed additional information: 65% in the physician group, and 59% in the chatbot group [125].

Patients might interact with the chatbot to detail their symptoms, and reciprocally, the chatbot could suggest an array of techniques for easing discomfort, such as breathing drills or relaxation methodologies. Moreover, chatbots have the capacity to dispatch medication alerts and tackle prevalent inquiries about palliative treatment alternatives, like palliative dialysis. Employing natural language processing, chatbots might partake in dynamic dialogues, tailoring their feedback to each patient’s distinct requirements and predilections [27,116,126]. Chatbots hold promise in guiding healthcare professionals in risk classification by tapping into data from EHRs and identifying patients with potential risks associated with specific treatment procedures [127]. Chatbots may determine whether patients would benefit from a meeting with a palliative care team, or create a comprehensive care plan to enhance the quality of life for patients with critical illnesses [20,128,129,130].

#### 5.4.2. Facilitating Communication and End-of-Life Decision Making

Chatbots stand well-poised to amplify communication efficacy by providing patients and their kin with precise and dependable data about prognosis, accessible treatment avenues, and prospective outcomes [79,126,131]. Patients might engage in dialogues with the chatbot to delve deeper into the advantages and potential pitfalls aligned with diverse treatment options [116,131,132]. In addition, chatbots could play a role in documenting patients’ inclinations concerning end-of-life care, revival, and medical maneuvers. Steering patients through these deliberations and ensuring the meticulous recording of their choices, chatbots play a part in enlightened decision making at pivotal junctures [81,133]. Moreover, chatbots can potentially assist the healthcare team in developing and refining effective communication skills within challenging situations such as end-of-life care and breaking bad news [134]. Chatbots might facilitate simulated case scenarios, refining language and tone in a risk-free environment, and give feedback. Consequently, this practice would enable better management of patients’ emotional responses and foster trust during critical moments [123].

#### 5.4.3. Providing Emotional Support and Resources

**Figure 8:**
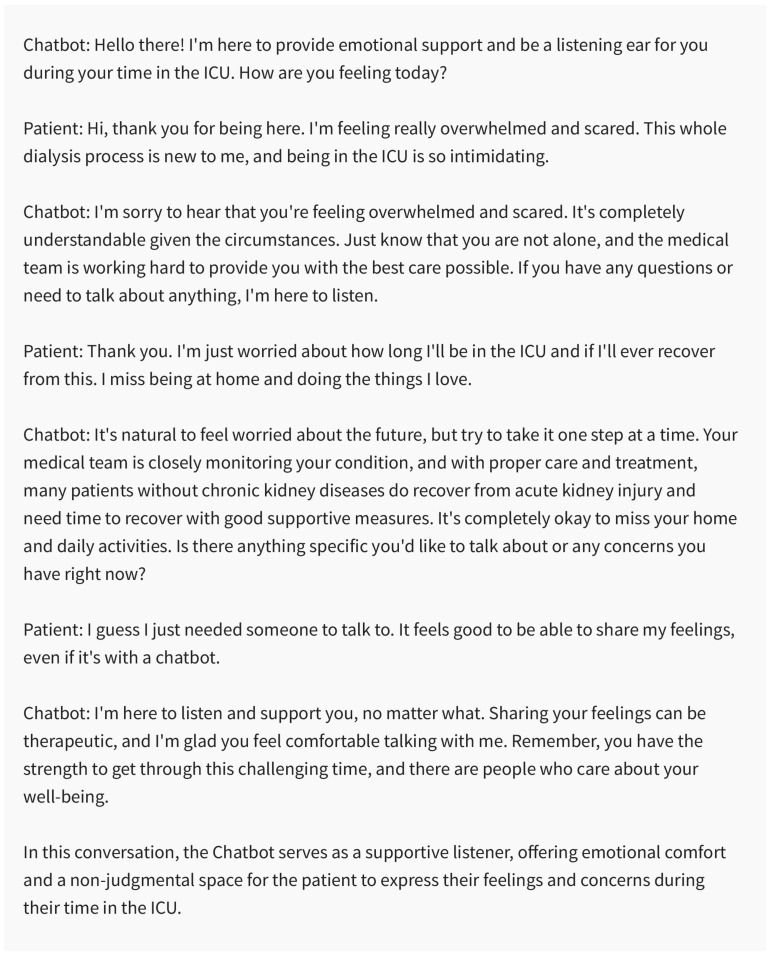
Chatbots serving as accessible sources of emotional support for patients. Created using ChatGPT 3.5.

Chatbots hold immense potential as readily available platforms, offering emotional support to patients and their families during the challenging phases of palliative care (Figure 8). They possess the ability to engage in active listening and voice interaction [68], detect patients’ concerns and emotional status through tone analysis [38,89], and give empathic responses [27,134,135]. Beyond that, chatbots might supply authenticated data and proffer resources connected to coping strategies, grief guidance [136], and community support groups [131,137].

Chatbots extend emotional support beyond the confines of physical healthcare settings by connecting patients and families with relevant resources and providing guidance. Table 3 illustrates the potential functions of chatbots in offering information to advance care plans for critically ill patients in the context of palliative nephrology scenarios.

Chatbots stand well-poised to act as virtual collaborators, allowing healthcare providers to navigate intricate and sensitive facets with heightened proficiency and empathy [138]. Chatbots can assist with navigating critical conversations, meaningful engagement, and support for patients and their families in the context of advance care planning and palliative care. Additionally, using chatbots could help alleviate emotional burden, reducing communication-related errors and misunderstandings, improving satisfaction, having a meaningful and positive impact on quality of life, and providing holistic care [126].

It remains vital to discern that, though chatbots might lend considerable support in palliative care, they are not meant to eclipse the innate human bond and individualized care rendered by medical experts [139]. Chatbots should be incorporated as complementary tools that enhance the services provided by palliative care teams, rather than seeking to replace them. Human oversight and intervention should be readily available to address complex situations and ensure patients receive appropriate care tailored to their circumstances. In comprehensive healthcare, chatbots play a significant role in offering valuable support. However, it is imperative to acknowledge that chatbots cannot act as substitutes for essential humanization and personalized doctor–patient interaction, which remains a crucial aspect of healthcare that professionals provide [139]. Instead, chatbots should be viewed as supplementary tools that augment services and alleviate the burden on the healthcare team. Particularly in intricate and demanding situations, the presence of healthcare professionals is indispensable, providing oversight and intervention to ensure patients receive holistic care tailored to their unique circumstances, with the warmth and compassion that only human beings can offer.

## 6. Future Directions of Chatbot Integration in Critical Care Nephrology

The integration of chatbots into care nephrology shows potential for improving patient care and outcomes. As this technology advances, there are areas where further development may potentially enhance the delivery of critical care nephrology services. One important area is decision support. By incorporating guidelines and evidence-based protocols, chatbots can potentially provide real-time recommendations for diagnosing, treating, and managing critical care nephrological conditions. This empowers healthcare providers to access up-to-date information and make decisions at the point of care, ultimately leading to better patient outcomes. Another promising avenue for chatbot integration is in analytics and risk stratification. Through the application of machine learning algorithms and predictive analytics, chatbots have the capability to thoroughly examine data, identifying individuals who may be at risk of experiencing complications or deteriorating health conditions. This proactive approach allows healthcare providers to intervene early, implement measures, and personalize treatment plans, ultimately optimizing patient outcomes. Furthermore, integrating chatbots with monitoring systems and wearable devices enables patient monitoring and remote care. This ensures that patients are under observation even when they are not physically present in a healthcare facility.

Integrating chatbots with remote monitoring systems and wearable devices enables continuous patient monitoring and remote care. By gathering real-time patient information, such as vital signs and fluid balance, chatbots have the ability to analyze these data and quickly alert healthcare professionals in case there are any abnormalities or changes in the patient’s condition. This approach to care improves safety, enables early intervention, and reduces the need for hospital readmissions.

To cater to a range of patients, future chatbots could potentially be designed to support communication and demonstrate cultural sensitivity. They may be able to effectively bridge language barriers by offering language options and adapting responses based on contexts. This inclusivity contributes to patient engagement, satisfaction, and overall health outcomes.

The fusion of chatbot technology with EHRs holds considerable promise for revolutionizing healthcare delivery. The integration could be a cornerstone for personalized, efficient, and streamlined care. As we progress with chatbot integration, it is important to address considerations regarding user privacy. Future developments should prioritize data security, confidentiality standards, and compliance with privacy regulations. The transparent functionality of chatbots combined with informed consent mechanisms helps build trust between patients, healthcare providers, and technology. Learning and improvement mechanisms play a role in optimizing the performance of chatbots. By using data from the literature, real patient data, and expert insights, chatbots can potentially be taught to continuously improve their algorithms, enhance their capabilities, and guarantee precision and applicability in the rapidly developing domain of critical care nephrology.

## 7. Conclusions

The integration of chatbots into critical care nephrology is an enticing and promising avenue to explore within the healthcare landscape. While their adoption remains uncommon, they offer numerous potential benefits, including improved accessibility to healthcare services, enhanced clinical decision making, and personalized patient education. However, it is imperative to address challenges associated with accuracy, data security, and technical integration to ensure the successful implementation of chatbots in this context. As technology progresses, incorporating chatbots in critical care nephrology can potentially become valuable in optimizing patient care and improving outcomes.

## Figures and Tables

**Table 1:** Strategies for addressing privacy and security concerns in chatbot implementation.

Challenges/Limitations	Suggestions and Implementation
Data privacy and confidentiality	- Implement robust encryption mechanisms
	- Adhere to data protection regulations (e.g., HIPAA, GDPR)
	- Use secure data storage practices
	- Employ strict access controls to prevent unauthorized breaches
Consent and transparency	- Obtain clear and informed consent from patients
	- Transparently explain data collection and usage
	- Provide privacy policies and consent forms
	- Enable patients to revoke consent or opt out of data sharing
Authentication and identity verification	- Implement robust authentication mechanisms
	- Use two-factor authentication or biometric verification
	- Establish secure user identification protocols
Secure integration with existing systems	- Conduct security assessments and vulnerability testing
	- Implement secure APIs and industry-standard protocols
	- Safeguard the transmission and storage of data
Ongoing monitoring and maintenance	- Regularly monitor chatbot activities
	- Implement robust logging mechanisms
	- Use intrusion detection systems
	- Keep the chatbot platform and infrastructure up to date
Staff training and awareness	- Train staff on data privacy and security best practices
	- Educate on secure coding, password management, and awareness of social engineering attacks
	- Foster a well-informed and vigilant workforce

HIPAA—Health Insurance Portability and Accountability Act; GDPR—General Data Protection Regulation; APIs—application programming interfaces.

**Table 2:** Example of chatbots providing recommendations for CRRT troubleshooting, prescription adjustment, and optimal duration of therapy.

Case Scenario 1: Alarms and Technical Difficulties	
Healthcare Provider	“I have encountered an alarm on the CRRT machine and require assistance in addressing it”.
Chatbot	“I can provide troubleshooting support for the alarm. Please follow these steps:
	
	1. Verify the integrity of the connections between the patient’s vascular access and the CRRT machine.
	2. Ensure proper alignment and absence of kinks in the tubing lines.
	3. Confirm correct attachment and sufficient volume of the dialysate and replacement fluid bags.
	4. Examine the machine’s display for error codes or messages and provide the specific details”.
Healthcare Provider	“The alarm message on the machine indicates ‘Low Blood Flow Rate.’”
Chatbot	“To resolve the low blood flow rate alarm, please undertake the following actions:
	
	1. Inspect the vascular access site for indications of clotting or obstruction.
	2. Adjust the pump speed within the prescribed range to increase the blood flow rate.
	3. Verify that the arterial and venous pressure monitoring lines are unobstructed and functioning correctly.
	4. If the issue persists, consider consulting a nephrologist for further evaluation”.
	
Case Scenario 2: Adjusting CRRT Prescription	
Healthcare Provider	“The patient’s clinical condition has deteriorated, and I believe adjustments to the CRRT prescription are necessary”.
Chatbot	“I can assist in modifying the CRRT prescription. Please provide the following information: Current patient weight and fluid balance. Latest laboratory results, including electrolyte levels and urea/creatinine clearance. Any significant changes in the patient’s hemodynamic stability or urine output”.




Healthcare Provider	“The patient has experienced a significant increase in fluid overload”.
Chatbot	“Considering the heightened fluid overload, you may contemplate the following modifications to the CRRT prescription: Increase the ultrafiltration rate to achieve the desired fluid removal. Adjust the dialysate composition to address specific electrolyte imbalances. Close monitoring of the patient for signs of hypotension or electrolyte disturbances during and after the adjustment is crucial. If needed, consult with a nephrologist for further guidance”.






Case Scenario 3: Determining Optimal Duration of CRRT	
Healthcare Provider	“The patient has been undergoing CRRT for several days, and I’m uncertain about the appropriate timing to discontinue the therapy”.
Chatbot	“I can offer guidance regarding the optimal duration of CRRT. Please provide the following information: Fluid balance of the patient over the past few days. Trends in kidney function, including serum creatinine and urine output. Overall clinical stability and any improvements in the patient’s condition”.




Healthcare Provider	“The patient’s fluid balance has improved, and kidney function is gradually recovering”.
Chatbot	“Considering the enhanced fluid balance and progressive kidney function recovery, the following factors may assist in determining the optimal duration of CRRT: Evaluate the patient’s ability to maintain sufficient urine output and electrolyte balance without CRRT. Assess the patient’s overall clinical stability and resolution of the underlying condition causing AKI. Monitor the patient’s kidney function trend closely, including serum creatinine levels and urine output. Determine the appropriateness of transitioning to alternative renal replacement therapies or supportive care measures”.


**Table 3:** Chatbot interventions for supportive care in palliative nephrology scenarios in critically ill patients.

Scenario	Chatbot Intervention
Patient with severe kidney injury and no dialysis option	The chatbot initiates a compassionate conversation, acknowledging the patient’s unique circumstances. It imparts information on alternative treatment modalities such as conservative management and palliative care. Moreover, the chatbot offers accessible resources, elucidating the advantages of symptom management, comfort measures, and supportive care. It underscores the significance of transparent communication with the healthcare team to address the patient’s needs and preferences adequately.
Patient on dialysis with poor prognosis and considering withdrawal	The chatbot provides a supportive and empathetic response, acknowledging the patient’s challenging decision. It imparts information on the potential benefits associated with withdrawing dialysis, focusing on comfort measures, and enhancing the patient’s overall quality of life. The chatbot emphasizes the importance of discussing this with the healthcare team, including nephrologists and palliative care specialists. It offers resources that provide comprehensive information about advance care planning, end-of-life discussions, and emotional support services. Additionally, the chatbot encourages the patient to involve their loved ones in the decision-making process, while reassuring them of the availability of healthcare professionals to address their concerns.
Family member seeking guidance on withdrawing dialysis	The chatbot engages in a compassionate conversation with the family member, acknowledging their concerns and emotions. It provides trustworthy information on various coping mechanisms, grief counseling services, and support groups tailored to individuals with kidney-related illnesses. The chatbot emphasizes the availability of healthcare professionals and encourages patients or family members to seek additional support as required. It offers links to resources to help them effectively navigate the emotional challenges associated with severe kidney injury and difficult treatment decisions.
Patient or family member seeking emotional support and resources	The chatbot offers a safe and empathetic platform for patients or family members to express their emotions and address their concerns. It provides trustworthy information on various coping mechanisms, grief counseling services, and support groups specifically tailored to individuals dealing with kidney-related illnesses. The chatbot emphasizes the availability of healthcare professionals and encourages patients or family members to seek additional support as required. It offers links to resources that can potentially help them effectively navigate the emotional challenges associated with severe kidney injury and difficult treatment decisions.
